# Semaphorin 7A promotes endothelial to mesenchymal transition through ATF3 mediated TGF-β2/Smad signaling

**DOI:** 10.1038/s41419-020-02818-x

**Published:** 2020-08-10

**Authors:** Lei Hong, Fengchan Li, Chaojun Tang, Ling Li, Lili Sun, Xiaoqiang Li, Li Zhu

**Affiliations:** 1 Department of Vascular Surgery, The Affiliated Drum Tower Hospital, Nanjing University Medical School, Nanjing, Jiangsu China; 2 Cyrus Tang Hematology Center, Collaborative Innovation Center of Hematology, Suzhou Key Laboratory of Thrombosis and Vascular Biology, Jiangsu Key Laboratory of Preventive and Translational Medicine for Geriatric Diseases, and State Key Laboratory of Radiation Medicine and Protection, Soochow University, Suzhou, Jiangsu China; 3 Department of Vascular Surgery, Anhui Provincial Hospital, University of Science and Technology of China, Hefei, Anhui China

**Keywords:** Epithelial-mesenchymal transition, Mesenchymal migration

## Abstract

Endothelial to mesenchymal transition (EndMT) is an important pathological change in many diseases. Semaphorin7A (Sema7A) has been reported to regulate nerve and vessel homeostasis, but its role in EndMT remains unclear. Here we investigate the effect of Sema7A on EndMT and the underlying mechanism. Sema7A-overexpressed human umbilical vein endothelial cells (Sema7A-HUVECs) were generated and showed lower levels of endothelial cell markers and higher levels of mesenchymal cell markers indicating the occurrence of EndMT. RNA-sequencing analysis showed a total of 1168 upregulated genes and 886 downregulated genes. Among them, most of the molecules associated with EndMT were upregulated in Sema7A-HUVECs. Mechanistically, Sema7A-HUVECs showed a higher TGF-β2 expression and activated TGF-β/Smad Signaling. Importantly, Sema7A overexpression upregulated activating transcription factor 3 (ATF3) that was found to selectively bind the promotor region of TGF-β2, but not TGF-β1, promoting TGF-β2 transcription, which was further confirmed by ATF3-siRNA knockdown approach. Blocking β1 integrin, a known Sema7A receptor, alleviated the expression of ATF3, TGF-β2, and EndMT in Sema7A-overexpressed HUVECs, implying a role of β1 integrin/ATF3/TGF-β2 axis in mediating Sema7A-induced EndMT. Using Sema7A-deficient mice and the partial carotid artery ligation (PCL) model, we showed that Sema7A deletion attenuated EndMT induced by blood flow disturbance in vivo. In conclusion, Sema7A promotes TGF-β2 secretion by upregulating transcription factor ATF3 in a β1 integrin-dependent manner, and thus facilitates EndMT through TGF/Smad signaling, implying Sema7A as a potential therapeutic target for EndMT-related vascular diseases.

## Introduction

During embryonic development and disease progression, endothelial cells (ECs) display a considerable plasticity of transition to other cell types, such as endothelial to mesenchymal transition (EndMT), in which the ECs lose specific endothelial markers such as CD31, VE-cadherin, and progressively express mesenchymal markers like α-SMA and fibroblast-specific protein-1 (FSP-1)^1^. With specific respect to atrioventricular canal and heart valve development, ECs in the region of the forming atrioventricular canal undergo EndMT to give rise to mesenchymal cells that form the endocardial cushion tissue and semilunar valves^2^. In the adult, EndMT has emerged as a player in the pathogenesis of chronic fibrotic injuries, such as cardiac fibrosis^1^, kidney fibrosis^3^, and system sclerosis^4,5^. EndMT-derived mesenchymal-like cells altered extracellular matrix collagen protein and matrix metalloproteinase (MMP) production, which contribute to fibrosis transition^6,7^. In the cardiovascular system, EndMT existed in atherosclerosis and is associated with plaque instability by altering collagen–MMP balance^8^. In addition, EndMT is involved in the pulmonary vascular remodeling, causing pulmonary arterial hypertension^9^. As EndMT is common in many pathological lesions, identification of key molecules involved in EndMT is highly demanded for the diagnosis and treatment of related diseases.

The semaphorin family contains a large number of secreted and membrane-bound proteins, which were originally detected on immunocyte membranes^10,11^. Semaphorin7A (Sema7A) was discovered based on sequence similarities with the vaccinia virus sema homolog A39R^12,13^, and signaling through plexins or integrins to exert functions^14^. Sema7A has been reported to associate with activity-dependent olfactory synapse formation, pulmonary fibrosis, multiple sclerosis, T-cell-mediated inflammatory responses, and breast tumor progression^15–19^. Whether Sema7A is involved in the progress of EndMT is unknown, although previous study reported that Sema7A promotes growth and migration of oral tongue squamous cell carcinoma by regulation epithelial–mesenchymal transition (EMT)^20^.

We recently shown that Sema7A promotes atherosclerosis by mediating endothelial dysfunction and monocyte–EC interaction^21^. As EndMT is common in atherosclerotic lesions^8^, we propose that Sema7A participates in the regulation of EndMT. In this study, we showed that Sema7A promotes EndMT through TGF/Smad signaling via β1 integrin/ATF3/TGF-β2 axis and that genetic deletion of Sema7A ameliorates EndMT induced by disturb flow (d-flow), implying a potential therapeutic strategy for EndMT-related diseases by targeting Sema7A/β1 integrin and downstream signaling molecules.

## Results

### Overexpression of Sema7A in HVUECs promotes EndMT

**Fig. 1:**
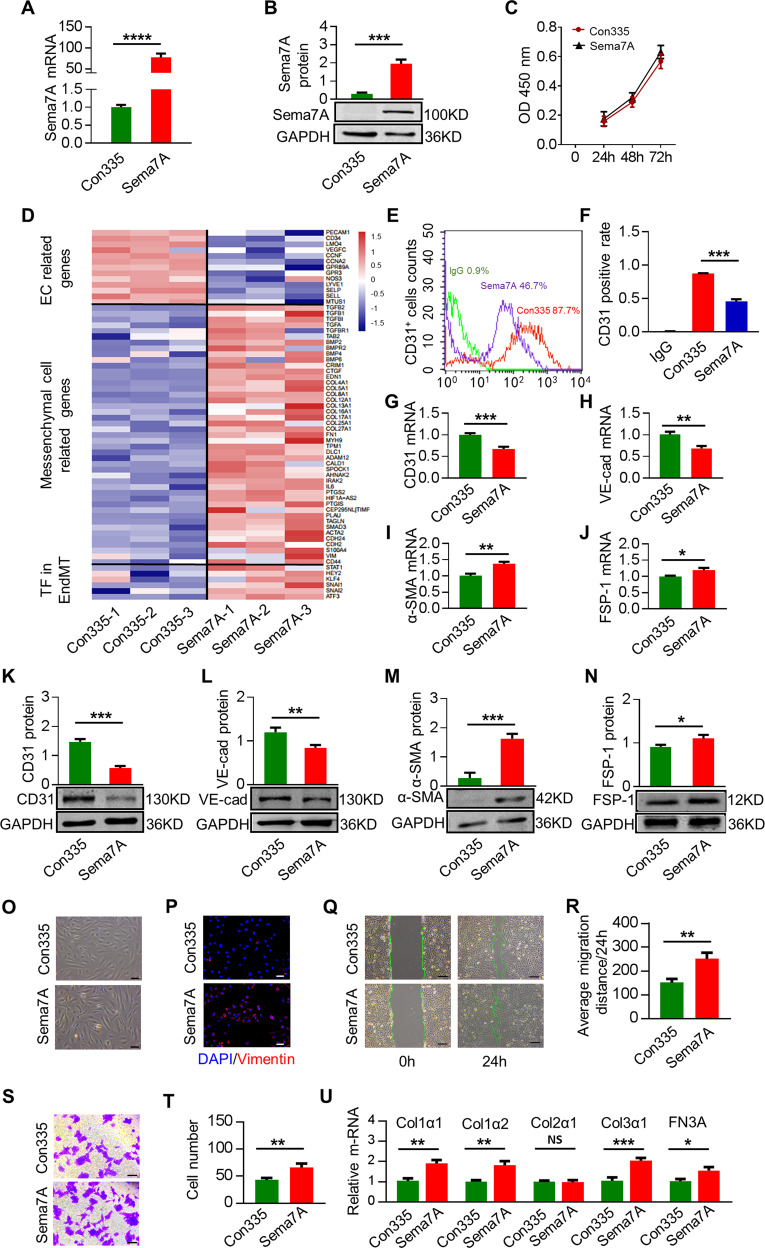
Overexpression of Sema7A in HUVECs promotes EndMT. **a** Sema7A mRNA expression in the Lenti-Con335-hSema7A-GFP-transduced HUVECs (Sema7A) was analyzed by quantitative polymerase chain reaction (qPCR) relative to Lenti-Con335-GFP-transduced control HUVECs (Con335). Data are mean ± SEM, *N* = 3, ****p < 0.0001. **b** Sema7A proteins in Sema7A-HUVECs were analyzed by western blotting normalized to GAPDH relative to Con335-HUVECs. Data are mean ± SEM, *N* = 3, ***p < 0.001. **c** There was no significant difference in cell proliferation activity between Con335-HUVECs and Sema7A-HUVECs indicated by CCK-8. **d** Heatmap shows different expression of endothelial cell (EC) related genes (top), mesenchymal cell related genes (middle), and transcription factor (TF) related to EndMT (bottom) for Con335-HUVECs and Sema7A-HUVECs (*p* < 0.05). **e**, **f** CD31^+^ cells in Con335-HUVECs and Sema7A-HUVECs were detected by a flow cytometer. Con335-HUVECs + IgG (green), Con335-HUVECs + CD31 antibody (red), Sema7A-HUVECs + CD31 antibody (purple) were indicated. Data are mean ± SEM, *N* = 3, ***p < 0.001. **g**–**j** CD31, VE-cadherin (VE-cad), α-SMA, and FSP**-**1 mRNA level were analyzed by qPCR normalized to GAPDH. Fold changes are shown. Data are mean ± SEM, *N* = 3, *p < 0.05; **p < 0.01; ***p < 0.001. **k**–**n** CD31, VE-cad, α-SMA, and FSP-1 proteins expression were analyzed by western blotting normalized to GAPDH. Data are mean ± SEM, *N* = 3. *p < 0.05; **p < 0.01; ***p < 0.001. **o** Con335-HUVECs and Sema7A-HUVECs morphology were observed by microscope. Scale bar = 50 µm. **p** Cells were immunofluorescently stained for DAPI (blue)/vimentin (red). Scale bar = 50 µm. **q**, **r** Con335-HUVECs and Sema7A-HUVECs were cultured and then subjected to wound healing assay. Phase contrast images are from the start of the assay (0 h) and after 24 h. Location of initial scratch margins indicated by dashed green lines. Scale bar = 100 µm. Data are mean ± SEM, *N* = 3, **p < 0.01. **s**, **t** Transwell cell invasion assay were used to detect the migration ability of cells. Representative images of transwell membranes with cells stained by crystal violet. Data are mean ± SEM, *N* = 3, **p < 0.01. **u** Col1α1, Col1α2, Col2α1, Col3α1, and FN3A mRNA level were analyzed by qPCR normalized to GAPDH. Fold changes are shown. Data are mean ± SEM, *N* = 3, *p < 0.05; **p < 0.01; ***p < 0.001. Collagen (Col), Fibronectin (FN).

Upregulation of Sema7A expression has been associated with various inflammatory diseases, including interstitial lung disease^22^, multiple sclerosis^23^, and rheumatoid arthritis^24^, in which EndMT is a common pathological change. Moreover, Sema7A functions as an effector molecule that induces pro-inflammatory cytokine production and the release of superoxide by monocytes/macrophages and neutrophils^15,25^, the pathological processes that promote EndMT. We therefore speculate that Sema7A is associated with EndMT. We generated Sema7A-overexpressed HUVECs (Sema7A-HUVECs) by transducing lentiviral vectors expressing human Sema7A with a green fluorescent protein (GFP) tag (Lenti-hSema7A-GFP) into primary HUVECs (Supplementary Fig. S1) with Lenti-con335-GFP-transduced HUVECs (Con335-HUVECs) as control. Sema7A expression were determined by quantitative real-time PCR (qPCR) and western blotting (Fig. 1a, b). Sema7A overexpression did not affect cell proliferation, as there was no difference in CCK-8 expression between Sema7A-HUVECs and Con335-HUVECs (Fig. 1c). We then performed an RNA-sequencing (RNA-seq) on Sema7A-HUVECs and Con335-HUVECs. Results showed that Sema7A overexpression caused the upregulation of a total of 1168 genes and downregulation of 886. Among them, most of the molecules associated with EndMT and mesenchymal cells were upregulated in Sema7A-HUVECs, as compared to Con335-HUVECs with a higher endothelial gene-expression profile (Fig. 1d).

We next examined the effect of Sema7A overexpression on the changes of markers of ECs and mesenchymal cells in Sema7A-HUVECs. Flow cytometry analysis revealed a 48% reduction of CD31^+^ cells in Sema7A-HUVECs (45.64 ± 2.76%) compared with con335-HUVECs control (87.48 ± 0.36%) (*p* < 0.001, *n* = 3) (Fig. 1e, f). Significantly, compared with con335-HUVECs, Sema7A-HUVECs presented lower levels of EC markers CD31 and VE-cadherin (VE-cad), and higher levels of mesenchymal cell markers α-SMA and FSP-1 as shown by qPCR (Fig. 1g–j) and western blotting (Fig. 1k–n). Next, the cell morphological observation showed that Sema7A-HUVECs became elongated and exhibited a spindle-like morphology, while Con335-HUVECs exhibited the characteristic cobblestone-like morphology (Fig. 1o). Vimentin is one of the protein markers of EndMT^26^. Through immunofluorescence staining, we detected vimentin expression in Sema7A-HUVECs but seldom in con335-HUVECs (Fig. 1p). Together, these results indicated that Sema7A overexpression induces EndMT. As the mobility of mesenchymal cells is higher than that of ECs, wound healing and transwell assays were performed to investigate the effect of Sema7A on EC migration. Results revealed that the ability of migration in Sema7A-HUVECs was significantly higher than that in Con335-HUVECs (Fig. 1q–t). The secretion of collagen is an important function of mesenchymal cells. Compared with con335-HUVECs, Sema7A-HUVECs presented higher levels of collagen 1α1, Collagen 1α2, Collagen 3α1, and Fibronectin 3A as shown by qPCR (1U).

### TGF-β2 gene expression and TGF-Smad signaling are augmented in Sema7A-HUVECs

**Fig. 2:**
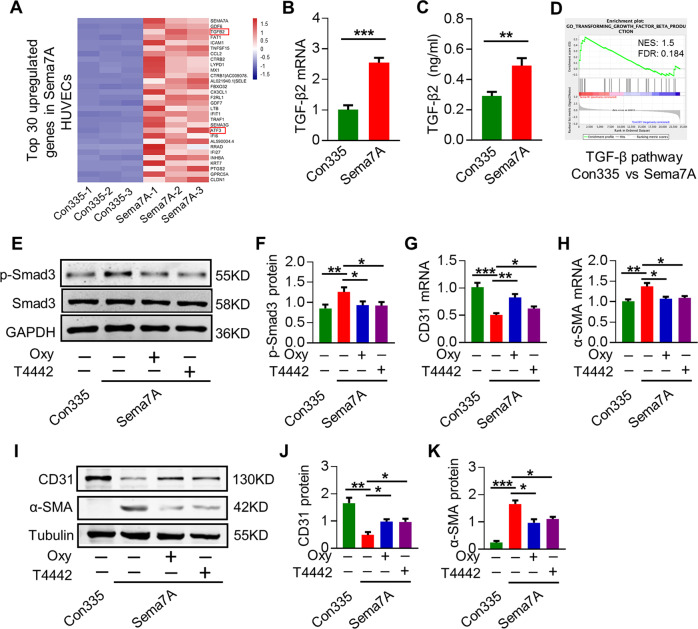
TGF-β2 gene expression and TGF-Smad signaling are augmented in Sema7A-HUVECs. **a** The top 30 upregulated genes in Sema7A-HUVECs compared with Con335-HVUECs. **b** TGF-β2 mRNA level was analyzed by qPCR normalized to GAPDH. Fold changes are shown. Data are mean ± SEM, *N* = 3, ***p < 0.001. **c** The concentration of TGF-β2 in cell supernatant was detected by ELISA. *N* = 10. Unpaired two-tailed Student’s *t* tests was used to analysis the data. Data are mean ± SEM, **p < 0.01. **d** GSEA based on gene ontology (GO) pathway database showed TGF-β signaling pathway was enrich in Sema7A-HUVECs. **e**, **f** Cells were treated with Oxymatrine (Oxy) (20 μmol/l) or T4442 (1 μg/ml) and the lysates were analyzed by western blotting for Smad3 phosphorylation, normalized to total Smad3. Data are mean ± SEM, *N* = 3, *p < 0.05; **p < 0.01. **g**, **h** CD31 and α-SMA mRNA in cells treated with inhibitors were analyzed by qPCR normalized to GAPDH. Fold changes are shown. Data are mean ± SEM, *N* = 3, *p < 0.05; **p < 0.01; ***p < 0.001. **i**–**k** CD31 and α-SMA proteins in cells treated with inhibitors were analysis by western blotting, normalized to tubulin. Data are mean ± SEM, *N* = 3, *p < 0.05; **p < 0.01; ***p < 0.001. T4442: TGF-β2 blocking antibody; Oxymatrine (Oxy): TGF/Smad signaling pathway inhibitor.

Among the 1168 upregulated genes in Sema7A-HUVECs, transforming growth factor-β2 (TGF-β2), a central regulator of fibrosis and interstitial changes in many diseases^27^, ranked in the top 30 (Fig. 2a), which led us to examine whether TGF-β2 is a potential candidate to mediate Sema7A-induced EndMT. By qPCR and ELISA, we showed that TGF-β2 was upregulated in Sema7A-HUVECs (Fig. 2b, c). Gene set enrichment analysis showed that TGF-β/Smad signaling pathway, a primary signaling pathway mediating EndMT, is enriched in Sema7A-HUVECs (Fig. 2d). We therefore assessed Smad3 phosphorylation (p-Smad3), an indicator of TGF-β/Smad signaling activity^28^ and showed that p-Smad3 was elevated in Sema7A-HUVECs. In contrast, inhibition of TGF/Smad signaling by inhibitor oxymatrine attenuated phosphorylation of Smad3 induced by Sema7A overexpression. Notably, blocking TGF-β2 by neutralizing antibody T4442 reduced phosphorylation of Smad3 as well, implying an upstream regulation of TGF-β2 on Smad3 in Sema7A-HUVECs (Fig. 2e, f). Moreover, inhibition of TGF/Smad signaling by oxymatrine or blocking TGF-β2 by antibody T4442 enhanced CD31 expression (Fig. 2g, i, j) and reduced α-SMA expression (Fig. 2h, i, k) in Sema7A-HUVECs. Together, our results suggest that Sema7A overexpression induced EndMT potentially by upregulating TGF-β2 and activating TGF-β/Smad signaling.

### Inhibition of ATF3 reduced TGF-β2 expression and Sema7A-induced EndMT

**Fig. 3:**
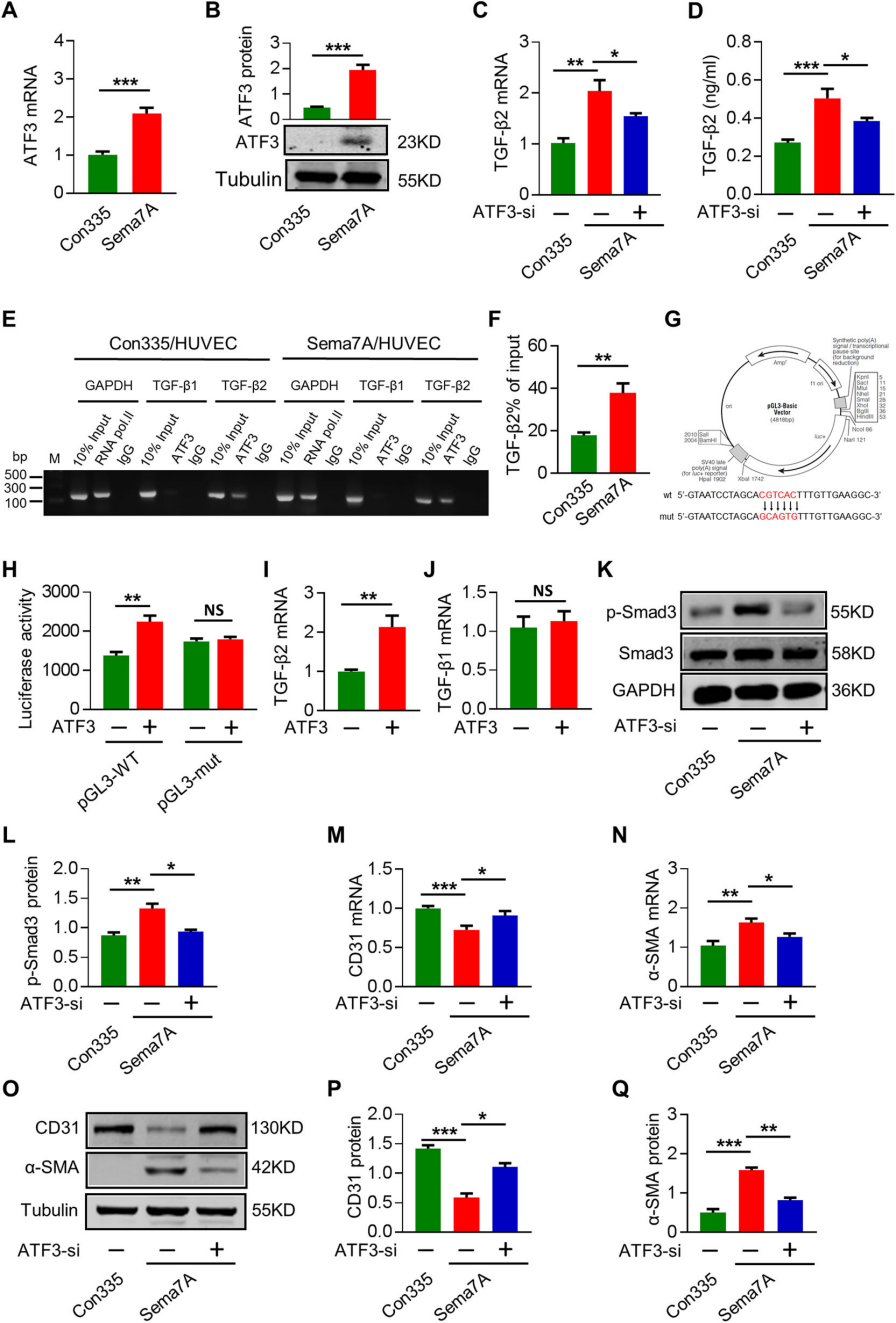
Inhibition of ATF3 reduced TGF-β2 expression and Sema7A-induced EndMT. **a** ATF3 mRNA level was analyzed by qPCR normalized to GAPDH. Fold changes are shown. Data are mean ± SEM, *N* = 3, ***p < 0.001 **b** ATF3 protein expression were analyzed by western blotting, normalized to tubulin. Data are mean ± SEM, *N* = 3, ***p < 0.001. **c** TGF-β2 mRNA level was analyzed by qPCR normalized to GAPDH. Fold changes are shown. Data are mean ± SEM, *N* = 3, *p < 0.05; **p < 0.01. **d** The concentration of TGF-β2 in cell supernatant was detected by ELISA among Con335-HUVECs, Sema7A-HUVECs, and Sema7A-HUVECs + ATF3-siRNA. *N* = 10. Data are mean ± SEM, *p < 0.05; ***p < 0.001. **e** Chip-qPCR product in agarose gel electrophoresis. **f** Chip-qPCR TGF-β2 percentage of input in con335-HUVECs and Sema7A**-**HUVECs were analyzed by qPCR normalized to IgG. Data are mean ± SEM, *N* = 3, **p < 0.01. **g** Schematic graph of the constructed reporter plasmid. TGF-β2 mut indicates the TGF-β2 mutation promoter region in ATF3 binding site. The mutated nucleotides in TGF-β2 fragments are in red letters. **h** Luciferase reporter assays were performed on HEK 293 T cells. Data are mean ± SEM, *N* = 3, **p < 0.01 vs negative control. **i**, **j** ATF3-overexpression plasmid was transfected to HUVECs, and the mRNA levels of TGF**-**β2 and TGF-β1 were performed by qPCR normalized to GAPDH. Fold changes are shown. Data are mean ± SEM, *N* = 3, **p < 0.01. **k**, **l** P-Smad3 protein in cells treated with siRNA was analyzed by Western blotting, normalized to total Smad3. Data are mean ± SEM, *N* = 3, *p < 0.05; **p < 0.01. **m**–**q** CD31 and α-SMA RNA and proteins in cells treated with siRNA or control was analyzed by qPCR and western blotting. Data are mean ± SEM, *N* = 3, *p < 0.05; **p < 0.01; ***p < 0.001.

Activating transcription factor 3 (ATF3) is an adaptive-response gene and its dysfunction associates with many cardiovascular diseases^29^. In addition to TGF-β2, RNA-seq analysis showed that ATF3 also ranked in the top 30 (Fig. 2a). The upregulation of ATF3 in Sema7A-HUVECs was verified through qPCR and western blotting (Fig. 3a, b). To ask whether ATF3 regulates TGF-β2 expression in Sema7A-induced EndMT, we introduced ATF3-siRNA to Sema7A-HUVECs (Supplementary Fig. S3) and showed that both TGF-β2 mRNA level and supernatant protein concentration were downregulated (Fig. 3c, d), implying an upstream regulation of ATF3 on TGF-β2 expression. Further study using Chip-qPCR assays showed a higher TGF-β2 percentage of input in Sema7A-HUVECs compared with Con335-HVUECs, while, TGF β1, another member of TGF family, was not pulled down by ATF3 antibody (Fig. 3e, f), indicating a selective binding of ATF3 to TGF-β2. Using luciferase reporter assay, we showed that luciferase activity of reporter gene containing wild-type TGF-β2 promoter (pGLC3-WT) was increased after ATF3 plasmid transfection, while a 6 bp mutation (−19 to −12, the upstream of transcriptional start site) of TGF-β2 promoter region in reporter gene (pGLC3-mut) eliminated this change (Fig. 3g, h). Furthermore, we observed that ATF3 overexpression in HUVECs upregulated TGF-β2, but not TGF-β1, mRNA expression (Fig. 3i, j).

We next investigated whether ATF3 participated in Sema7A-induced EndMT by regulating TGF-β2 expression. As TGF-β2 activated TGF/Smad signaling to induce EndMT in Sema7A-HUVECs, we transfected Sema7A-HUVECs with ATF3-siRNA and found that Smad3 phosphorylation was reduced (Fig. 3k, l), indicating that inhibiting ATF3 reduced TGF-β/Smad signaling. Using the same strategy, we showed that inhibition of ATF3 upregulated CD31 and downregulated α-SMA, indicating an alleviation of EndMT (Fig. 3m–q). Together, our data suggest that the Sema7A overexpression enhances ATF3 expression that in turn promotes TGF-β2 transcription, inducing EndMT.

### β1 integrin mediates Sema7A signal to TGF-β2 via ATF3

**Fig. 4:**
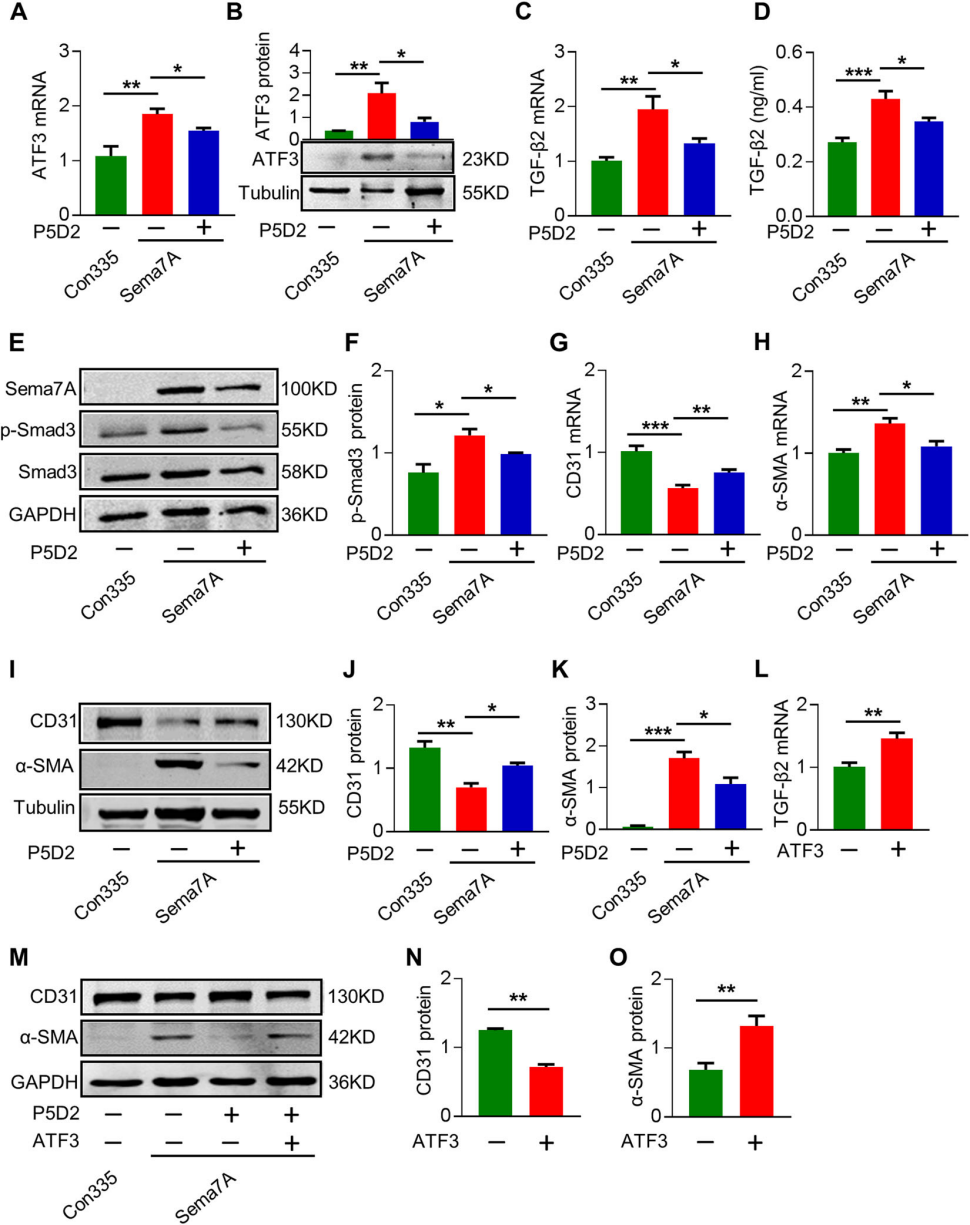
Β1 integrin mediates Sema7A signal to TGF-β2 via ATF3. **a** Cells were treated with β1 integrin antibody (P5D2), and ATF3 mRNA level was analyzed by qPCR normalized to GAPDH. Fold changes are shown. Data are mean ± SEM, *N* = 3, *p < 0.05; **p < 0.0.1. **b** ATF3 protein expression was analyzed by western blotting, normalized to tubulin. Data are mean ± SEM, *N* = 3, *p < 0.05; **p < 0.01. **c** TGF-β2 mRNA level was analyzed by qPCR normalized to GAPDH. Fold changes are shown. Data are mean ± SEM, *N* = 3, *p < 0.05; **p < 0.0.1. **d** The concentration of TGF-β2 in cell supernatant was detected by ELISA for Con335-HUVECs, Sema7A-HUVECs, and Sema7A-HUVECs + P5D2. Data are mean ± SEM, *N* = 10, *p < 0.05; ***p < 0.001. **e**, **f** Smad3 phosphorylation were analyzed by western blotting, normalized to total Smad3. Data are mean ± SEM, *N* = 3, *p < 0.05. **g**, **h** CD31 and α-SMA mRNA level were analyzed by qPCR normalized to GAPDH. Fold changes are shown. Data are mean ± SEM, *N* = 3, *p < 0.05; **p < 0.01; ***p < 0.001. **i**–**k** CD31 and α-SMA proteins were analyzed by western blotting, normalized to tubulin. Data are mean ± SEM, *N* = 3, *p < 0.05; **p < 0.01; ***p < 0.001. **l** ATF3 overexpression plasmid was transfected into P5D2 incubated Sema7A-HUVECs, and TGF-β2 mRNA expression in Sema7A-HUVECs + P5D2 and Sema7A-HUVECs + P5D2 + ATF3 were analyzed by qPCR normalized to GAPDH. Fold changes are shown. Data are mean ± SEM, *N* = 3, **p < 0.01. **m**–**o** CD31 and α-SMA protein expressions were analyzed by western blotting, normalized to GAPDH. Data are mean ± SEM, *N* = 3, **p < 0.01 (Sema7A-HUVECs + P5D2 vs Sema7A-HUVECs + P5D2 + ATF3). P5D2 β1 integrin antibody.

β1 integrin and Plexin C1 are known Sema7A receptors^14,30^. β1 integrin regulates EC–cell junctions, changes in the cytoskeleton, and matrix adhesion, notably induces EC destabilization^31,32^. To investigate the role of β1 integrin in Sema7A-induced EndMT, we treated Sema7A-HUVECs with a β1 integrin blocking monoclonal antibody (P5D2), and found that β1 integrin blockage reduced Sema7A-induced increase of ATF3 and TGF-β2 (Fig. 4a–d), Smad3 phosphorylation (Fig. 4e, f), and EndMT as indicated by upregulated CD31 and downregulated α-SMA in Sema7A-HUVECs (Fig. 4g–k). To confirm the role of β1 integrin and ATF3 axis in mediating Sema7A-induced EndMT, we transfected ATF3 plasmid into Sema7A-HUVECs that were preincubated with P5D2 to block β1 integrin activation by Sema7A. Results showed that ATF3 overexpression rescued TGF-β2 expression (Fig. 4l) and EndMT (Fig. 4m–o) pre-inhibited by β1 integrin blockage as shown by the reduced CD31 expression and enhanced α-SMA expression. These results indicated that Sema7A promotes EndMT through integrin β1 integrin-ATF3 axis.

### Loss of Sema7A reduces EndMT in vivo

**Fig. 5:**
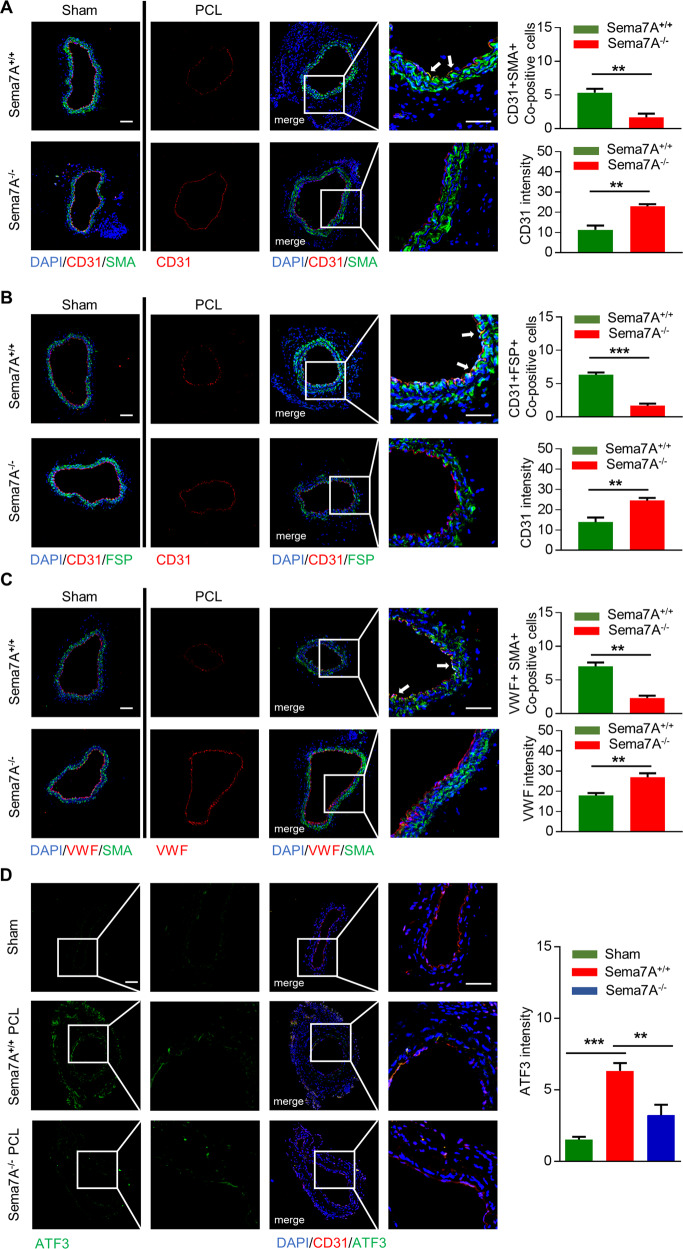
Loss of Sema7A reduces EndMT in vivo. Carotid artery exposed to d-flow from Sema7A^+/+^ and Sema7A^−^^/−^ mice were immunofluorescently stained for various endothelial–mesenchymal marker combinations as indicated (**a**) CD31 (red)/α-SMA (green), (**b**) CD31 (red)/FSP-1 (green), (**c**) VWF (red)/α-SMA (green), and DAPI (blue) 1 week after PCL, scale bar = 50 µm. The first column is the sham group, the second column is the single channel stained with CD31 or VWF, the third column is the merge channel stained with CD31 + SMA, CD31 + FSP, or VWF + SMA, and the fourth column is the enlarged image of the box in the third column. Co-positive cells per × 60 field were counted and shown on the right (upside). Quantification of CD31 or VWF fluorescence intensity of intima is shown on the right (downside). Data are mean ± SEM, **p < 0.01; ***p < 0.001. **d** Carotid artery exposed to d-flow from Sema7A^+/+^ and Sema7A^−^^/−^ mice were immunofluorescently stained for ATF3 (green) and CD31 (red). The first column is the single channel stained with ATF3, the second column is the enlarged image of the box in the first column, the third column is the merge channel (CD31 + ATF3), and the fourth column is the enlarged image of the box in the third column. Quantification of ATF3 fluorescence intensity of intima is shown on the right. Data are mean ± SEM, **p < 0.01; ***p < 0.001.

Our previous study reported that d-flow induced by partial carotid ligation (PCL) model increases the expression of Sema7A in vascular ECs^33^. Thus, we used the PCL model to investigate the effect of Sema7A on EndMT in vivo. During EndMT, ECs in intermediate stages of EndMT express both EC and mesenchymal cell markers. In wild-type (WT) mice, co-localization of CD31/α-SMA, CD31/FSP-1, or VWF/α-SMA were observed in the endothelium of the carotid artery upon PCL by immunofluorescence staining, while in Sema7A-deficient mice, co-localization of CD31 with α-SMA or FSP-1 was hardly seen (Fig. 5a–c). The total fluorescence intensity analysis showed a reduction of CD31 and VWF signal in the endothelium of the carotid artery upon PCL in WT mice, compared with Sema7A-deficient mice (Fig. 5a–c). These results suggest that Sema7A expression promotes EndMT in vivo. In consistent, compared with sham group, disturbed flow upregulated ATF3 expression in Sema7A^+/+^ mice, while Sema7A deletion mitigated this change (Fig. 5d).

## Discussion

In this study, we showed that the expression of endothelial Sema7A induces EndMT. Sema7A upregulates ATF3 in a β1 integrin-dependent manner and in turn promotes TGF-β2 transcription. Secreted TGF-β2 activates TGF-β2/Smad signaling pathway and subsequently induces EndMT. Sema7A deficiency mitigates EndMT induced by d-flow in vivo.

In the canonical signaling pathway of EndMT, TGF-β binds to the membrane receptors and phosphorylates the receptor-associated Smad proteins (Smad2 and Smad3). Smad2/3 interacts with Smad4 and translocates into the nucleus to initiate Smad-mediated transcriptional events, including EMT, EndMT, fibrosis, and others^34–36^. The TGF-β superfamily contains 33 members that can be subclassified into several subfamilies, including TGF-βs (TGF-β1, -β2, and -β3), bone morphogenetic proteins, growth differentiation factors, activins, inhibins, nodal, and anti-mullerian hormone proteins^37^. Both TGF-β1 and TGF-β2 can induce EndMT^8,38,39^. Previous studies demonstrated that TGF-β1 promotes the secretion of Sema7A and induces pulmonary fibrosis and multiple sclerosis^16,18,40^. Whether TGF-β2 plays a similar role in Sema7A expression is unknown. Here we show that Sema7A overexpression enhances TGF-β2, but not TGF-β1, secretion. How Sema7A overexpression enhances TGF-β2 is unknown. Previous study showed that ATF3 is necessary for TGF-β1 to upregulate its target genes, such as snail, slug, and twist, which induces EMT and EndMT, and ATF3 forms a positive-feedback loop for TGFβ1^41^. However, we show here that upregulated ATF3 enhances TGF-β2, but not TGF-β1, secretion. The reason for this selectivity is potentially due to the CRE/ATF element (GCACGTCA) in TGF-β2 promoter that TGF-β1 lacks^42^.

ATF3 is a member of the CREB/ATF family, participating in the development of cancer^43,44^, atherosclerosis, hypertension, and ischemic heart diseases^29^. ATF3 exhibits a low expression level in quiescent cells, but its expression increases under stress conditions, such as injury, ischemia, or ischemia/reperfusion^29^. We found that Sema7A overexpression upregulates transcription factor ATF3 that enhanced downstream TGF-β2 secretion, mediating ECs dysfunction and EndMT in Sema7A overexpression HUVECs. Meanwhile, ATF3 were found to regulate multiple targets genes and affect numerous cell types^29^. The effect of ATF3 on ECs is controversial, some reported that ATF3 protects ECs from apoptosis induced by TNF-a or inhibits pro-inflammatory gene-vascular cell adhesion molecule 1 expression^45,46^. However, others showed that ATF3 promotes ECs death in atherosclerosis, LPS induced inflammatory responses, and pro-inflammatory genes-E-selection and intercellular cell adhesion molecule 1 expression^47–49^. The puzzling effects of ATF3 on ECs may lie in different transcription regulation in response to different stimuli. Therefore, the interpretation for the role of ATF3 in mediating the effect of Sema7A on EndMT should be cautious and further investigation is demanded.

β1 integrin is the major receptor of Sema7A on ECs, and its activation promotes endothelial destabilization^31^. Our results showed that blocking β1 integrin prevents EndMT of ECs induced by Sema7A overexpression, which is consistent with its function on ECs as previously reported^31^. Moreover, ATF3 overexpression reversed the β1 integrin antibody (P5D2) inhibitory effect on EndMT, suggesting that β1 integrin-ATF3 axis transmits Sema7A signal to downstream TGF/Smad pathway to induce EndMT. Further studies are warranted to explore how ATF3 mediates Sema7A/β1 integrin outside-in signal.

**Fig. 6:**
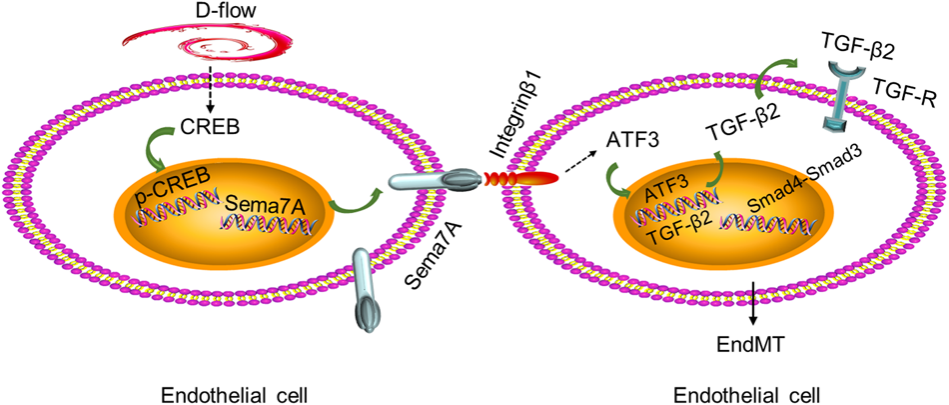
A proposed model for the role of Sema7A in EndMT. When exposed to d-flow, endothelial Sema7A expression is upregulated, potentially by the cAMP/CREB (cAMP response element-binding protein) pathway. Endothelial Sema7A upregulates the transcription factor ATF3 through interaction with β1 integrin, leading to TGF-β2 transcription, and activation of TGF/Smad3 signaling pathway, and thus inducing EndMT.

In summary, our results indicate that Sema7A upregulates transcription factor ATF3 through membrane receptor β1 integrin, leading to enhanced TGF-β2 transcription and activation of TGF/Smad3 signaling pathway, and thus inducing EndMT (Fig. 6). Since Sema7A is involved in atherosclerosis^33^, pulmonary fibrosis^16^, liver fibrosis^50^, and multiple sclerosis pathogenesis^18^ that are associated with EndMT^5,8,35,51,52^, our findings from both Sema7A-overexpressed cells and Sema7A-deficient mice provide a clue that Sema7A-β1 integrin and downstream signaling molecules could be regarded as promising targets for treatment of diseases associated with EndMT.

## Materials and methods

### Reagents and animals

Oxymatrine was purchased from MedChemExpress (MedChemExpress, New Jersey, USA Cat#16837-52-8). TGF-β2 polyclonal antibody was purchased from Sigma (Sigma, St. Louis, USA, Cat#T4442). β1 integrin neutralizing antibody P5D2 was purchased from Abcam (Abcam, UK, Cat#24693). The primary antibodies used in this study include mouse anti-human α-SMA (Abcam, UK, Cat#32575), rabbit anti-human CD31(Abcam, UK, Cat#28364), rabbit anti-human FSP-1 (Abcam, UK, Cat#41532), mouse anti-GAPDH (Abcam, UK, Cat#181602), rabbit anti-mouse VWF (Abcam, UK, Cat#6994), mouse anti-mouse fibronectin (Abcam, UK, Cat#6328), mouse anti-human vimentin (Abcam, UK, Cat#8069), rabbit anti-human Sema7A (Thermo Fisher Scientific, Waltham, USA, Cat#PA5-28971), rabbit anti-human ATF3 (CST, Danvers, USA, Cat#33593), rabbit anti-human VE-cadherin (CST, Danvers,USA, Cat#2500), mouse anti-β tubulin (CST, Danvers, USA, Cat#2146s), rabbit anti-human p-Smad3 (BOSTER, China, Cat#BM4033), rabbit anti-human Smad3 (BOSTER, China, Cat#BM3919), ATF3 (Novus Biologicals, USA, Cat# 85816), and CD31 antibody (BD Biosciences, New Jersey, USA, Cat#555446). The secondary antibodies include Alexa Fluor donkey anti-rabbit488, Alexa Fluor rabbit anti-mouse 555, Alexa Fluor donkey anti-mouse 647 (Abcam, UK, Cat#150077, Cat#150118), DAPI (Abcam, UK, Cat#ab104139).

Lentiviral vectors expressing human Sema7A-GFP (Lenti-Con335-hSema7A-GFP) and control con335-GFP (Lenti-Con335-GFP), ATF3 plasmid, and siRNA were purchased from GeneChem company (Genechem, China).

Primary human umbilical vein endothelial cells (HUVECs) (ATCC, Maryland, USA, Cat# PCS-100-010) were maintained in vascular cell basal medium (ATCC, Maryland, USA, Cat#PCS-100-030) containing ascorbic acid (ATCC, Maryland, USA, Cat#PCS-999-006), FBS (ATCC, Maryland, USA, Cat#PCS-999-010), rhEGF (ATCC, Maryland, USA, Cat#PCS-999-018), heparin sulfate (ATCC, Maryland, USA, Cat#PCS-999-011), L-glutamine (ATCC, Maryland, USA, Cat#PCS-999-017), rhVEGF (ATCC, Maryland, USA, Cat#PCS-999-024), rhFGF-b (ATCC, Maryland, USA, Cat#PCS-999-020), rhIGF-1 (ATCC, Maryland, USA, Cat#PCS-999-021), and hydrocortisone (ATCC, Maryland, USA, Cat#PCS-999-014).

Sema7A^−/−^ mice on C57BL/6 background were purchased from Jackson Laboratories (Bar Harbor, ME, USA) and housed in a specific pathogen-free facility. All experiments were approved by the University Committee on Animal Care of Soochow University (20140431). After experiments, the animals were euthanized by CO_2_ inhalation.

### Generation of Sema7A-overexpressed HUVECs (Sema7A-HUVECs)

Lenti-con335-hSema7A-GFP was added into HUVECs at a multiplicity of infection of 10:1 in serum-free medium. The lenti-virus containing con335 backbone (Lenti-con335-GFP) was used as a negative control. Sema7A expression was observed by positive green fluorescence under a laser confocal microscope (IX-81, Olympus, Japan) and verified by qPCR and Western blotting 72 h after the transduction. Vector information and sequences are listed in Supplementary Fig. S1.

### Generation of ATF3 overexpressed or knockdown HUVECs

ATF3 plasmid or siRNA was transfected into HUVECs using Lipofectamine 3000 (GeneChem, China) according to the manufacturer’s instructions, and backbone con335 or con207 was transfected as negative controls. Cells were collected at 48 h after transfection, and the expression of mRNA and protein was confirmed by qPCR and western blotting. Vector information and sequences are listed in Supplementary Figs. S2 and S3.

### RNA isolation and qPCR

RNA was isolated from cultured cells, using RNA simple Total RNA Kit (Tiangen, Biotech, China). RNA was quantified with NanoDrop 2000 spectrophotometer (Thermo Fisher Scientific, Waltham, USA). Isolated RNAs were reverse transcribed into cDNAs, using 5×All-In-One RT Master Mix (abm Canada, Cat#G490). Reaction conditions were as follows: 25 °C for 5 min, 42 °C for 30 min, 85 °C for 5 min, and finished at 4 °C. Then qPCR was carried out using the SYBR Green FastMix Reaction Mixes kit (Roche, Switzerland) in a real-time-PCR System (Roche, Switzerland, Cat#LightCycler 480). RNA expression was analyzed using the 2^−ΔΔCT^ methods. Primer sequences are listed in Supplementary Table 1.

### Microarray analysis

Total RNA was isolated from HUVECs. RNA quality was assessed in Bioanalyzer 2100 (j06-02 Agilent). Gene-expression profiling was performed using KAPA SYBR FAST Master Mix, DNA quantification standards, and primer premix kit (KAPA Biosystem, Roche, Switzerland, Cat#KK4602, KK4808). All procedures are executed by the capitalbio company.

### Immunoblotting

Cells were lysed on ice using RIPA lysis buffer (1% Triton X-100, 1% deoxycholate, 0.1% SDS, 10 mM Tris and 150 mM NaCl) with protease and phosphatase inhibitor cocktail (Santa Cruz Biotechnology Inc, Heidelberg, Germany). After lysis, cell lysates were split by ultrasonic (Sonics, China, Cat#VCX130) and centrifuged at 14,000 *g* for 5 min at 4 °C. The protein concentration of supernatant was measured using a BCA protein assay kit (Beyotime, China). The reduced proteins (30 μg) in 4 × sample buffer (Invitrogen) and β-2-mercaptoethanol were heated at 95 °C for 5 min before loading. Protein samples were separated on 10% gel and transferred to nitrocellulose filter membranes. The membranes were blocked with 5% non-fat dry milk (Bio-Rad, Calif, USA) in TBS-T, and incubated with primary antibody overnight at 4 °C, followed by the fluorescent secondary antibodies for 1 h at room temperature. After washing, membranes were scanned using the Odyssey infrared imaging system (LI-COR Biosciences, USA). Densitometric analysis was done using Image J software (NIH) to quantify protein expression levels with GAPDH or tubulin as internal control.

### Evaluation of cell growth and viability

CCK-8 was used to measure the cell growth and viability. Briefly, HUVECs were seeded into 96-well plates (1 × 10^3^ cells/well). For successive 3 days, 10 μl CCK-8 was added, and the absorbance at 450 nm was detected by using Epoch (Bio-Tek, VT, USA). The data were statistically analyzed and exhibited by the growth curves.

### Flow cytometry

Con335-HUVECs and Sema7A-overexpressed HUVECs were detached from petri-dish and stained with an anti-human CD31 antibody (1 μg/ml) for 30 min at room temperature. After PBS washing, cells expressing CD31 were analyzed by a flow cytometer (BD Biosciences, New Jersey, USA).

### **Scratch wound assay**

Con335-HUVECs and Sema7A-HUVECs were seeded in 6-well plates (3 × 10^5^ cells/well). Cells were starved with serum-free medium for 12 h before a scratch wound assay was carried out and a 200-µl pipette tip was used to create a linear scratch at nearly 95% confluence. Subsequently, fresh medium with FBS was added. HUVECs were grown for 24 h and images were captured at 0 h and 24 h, using a Nikon Microscope. Closure of the wound area was quantitated by Image-Pro Plus software. The total area of the blank region was measured and the average distance was obtained by dividing the total area by the height. Data are summarized as means ± standard error of the mean (SEM).

### Transwell assay

Cells were plated in the upper chamber of the BD BioCoat chamber (BD, USA, 353097) with serum-free medium. The migrated ECs toward a gradient of 20% FBS in the lower chamber was monitored. After incubation for 24 h, the cells on the upper surface of the membrane were removed and cells located on the lower surface of the membrane were fixed in 4% PFA for 20 min and then stained with crystal violet for 5 min. Images of invasion cells were obtained using an inverted microscope. The invasion assay analysis was performed using Image J software and GraphPad Prism software.

### Immunostaining

Cells were fixed with 4% paraformaldehyde and blocked with immunostaining Blocking Buffer (Beyotime, China, P0102). Immunostaining was performed using primary antibody and DAPI. After incubation with fluorescent-labeled secondary antibody. Images were acquired using a confocal microscope under the same conditions for each experiment.

### ELISA

Commercially human TGF-β2 ELISA kit (Abcam, UK, Cat#ab100648) was used to measure TGF-β2 in the supernatant of cell cultures according to the manufacturer’s instructions.

### Chromatin immunoprecipitation ChIP-qPCR

HUVECs were fixed in plates with 1% formaldehyde and cross-linked protein–DNA complexes were prepared with EZ-ChIP^™^ (Merck Millipore). Equal volumes of protein–DNA complex from each sample were mixed with primary antibodies ATF3 with gentle rotation at 4 °C for 18 h. Magnetic beads were added and rotated for additional 2 h. Immunoprecipitates were eluted from the beads and treated with protease K to digest protein. DNA was then purified according the manufacturer’s instructions and analyzed in qPCR assays, using primers flanking the ATF3 binding sites on human TGF-β2 promoters. The PCR primers for human TGFβ-2 are (5′ to 3′): forward—CCTCCTTCCTCCCTTACCC, reverse—TCTCTGAACCACGTGTCTGC. All samples were run in triplicates, and signals were normalized to preserve input control DNA.

### Luciferase assays

The luciferase reporter vectors pGL3-Basic vector with both wild-type or mutated TGFβ-2 promoter region were constructed. After digestion by Xhol and HindIII, the fragments of wild-type and mutant TGFβ-2 promoter region were cloned into the Xhol and HindIII sites of reporter luciferase vector (Applied Biosystems, USA) and named as pGL3/WT and pGL3/mut, respectively. Human embryonic kidney cell line 293 T (HEK 293 T) cells were seeded in 96-well plates 12 h prior to transfection. In each well, cells were transfected with 0.1 μg reporter plasmids, together with 0.1 μg pcDNA3.1-vector containing ATF3 plasmid or empty vector as control. Transfection was performed using Lipofectamine 3000. Assay was carried out by the dual luciferase reporter assay system (Promega, Wisconsin, USA, Cat#E1500).

### Partial carotid artery ligation

PCL was performed as previously reported by others^22,31,33^. Briefly, mice were anesthetized with 1% pentobarbital sodium (Merck Millipore, Germany, Cat#57-33-0,). A cervical midline incision of 4–5 mm was made in the neck. Left common carotid artery (LCA) was exposed by blunt dissection. Three of four caudal branches of LCA (left external carotid, internal carotid, and occipital arteries) were ligated with 10–0 silk suture. After closing the incision, the animals were maintained on a heating pad until they regained consciousness. The diagrammatic drawing is in Supplementary Fig. S4.

### Immunostaining

The samples were fixed in 4% paraformaldehyde overnight and washed with PBS 5 min for three times before dehydrating with 20% sucrose. Next day, tissues were embedded in optimal cutting temperature compound and stored at −80 °C. Frozen section (8 μm) were incubated with primary antibodies or an IgG control at 4 °C overnight, fluorescent secondary antibodies the next day for 1 h and DAPI 15 min. Images were filmed using a multicolor digital camera on an IX-81 laser confocal microscope (Olympus, Japan).

### Statistical analysis

Data were analyzed by Prism 7.0 GraphPad software. All the data are presented as mean ± SEM. Unpaired two-tailed Student’s *t* tests, Mann–Whitney *U* test, and one-way ANOVA were used for data analysis followed by post hoc test. *p* < 0.05 was considered statistically significant.

## Supplementary information

supplemental table 1

supplemental figure legends

supplemental figure 1

supplemental figure 2

supplemental figure 3

supplemental figure 4

## References

[1] Zeisberg EM (2007). Endothelial-to-mesenchymal transition contributes to cardiac fibrosis. Nat. Med..

[2] Kisanuki YY (2001). Tie2-Cre transgenic mice: a new model for endothelial cell-lineage analysis in vivo. Dev. Biol..

[3] Zeisberg EM, Potenta SE, Sugimoto H, Zeisberg M, Kalluri R (2008). Fibroblasts in kidney fibrosis emerge via endothelial-to-mesenchymal transition. J. AM. Soc. Nephrol..

[4] Taniguchi T (2015). Fibrosis, vascular activation, and immune abnormalities resembling systemic sclerosis in bleomycin-treated Fli-1-Haploinsufficient mice. Arthritis Rheumatol.

[5] Manetti M (2017). Endothelial-to-mesenchymal transition contributes to endothelial dysfunction and dermal fibrosis in systemic sclerosis. Ann. Rheum. Dis..

[6] LeBleu VS (2013). Origin and function of myofibroblasts in kidney fibrosis. Nat. Med..

[7] Wu J (2016). The origin of matrix-producing cells that contribute to aortic fibrosis in hypertension. Hypertension.

[8] Evrard SM (2016). Endothelial to mesenchymal transition is common in atherosclerotic lesions and is associated with plaque instability. Nat. Commun..

[9] Ranchoux B (2015). Endothelial-to-mesenchymal transition in pulmonary hypertension. Circulation.

[10] Pasterkamp RJ, Kolodkin AL (2003). Semaphorin junction: making tracks toward neural connectivity. Curr. Opin. Neurobiol..

[11] Kruger RP, Aurandt J, Guan KL (2005). Semaphorins command cells to move. Nat. Rev. Mol. Cell Biol..

[12] Delorme G, Saltel F, Bonnelye E, Jurdic P, Machuca-Gayet I (2005). Expression and function of semaphorin 7A in bone cells. Biol. Cell.

[13] Liu H (2010). Structural basis of semaphorin-plexin recognition and viral mimicry from Sema7A and A39R complexes with PlexinC1. Cell.

[14] Pasterkamp RJ, Peschon JJ, Spriggs MK, Kolodkin AL (2003). Semaphorin 7A promotes axon outgrowth through integrins and MAPKs. Nature.

[15] Suzuki K (2007). Semaphorin 7A initiates T-cell-mediated inflammatory responses through alpha1beta1 integrin. Nature.

[16] Kang H, Lee CG, Homer RJ, Elias JA (2007). Semaphorin 7A plays a critical role in TGF-β1–induced pulmonary fibrosis. J. Exp. Med..

[17] Inoue N, Nishizumi H, Naritsuka H, Kiyonari H, Sakano H (2018). Sema7A/PlxnCl signaling triggers activity-dependent olfactory synapse formation. Nat Commun.

[18] Eixarch H, Gutiérrez-Franco A, Montalban X, Espejo C (2013). Semaphorins 3A and 7A: potential immune and neuroregenerative targets in multiple sclerosis. Trends Mol. Med..

[19] Black SA, Nelson AC, Gurule NJ, Futscher BW, Lyons TR (2016). Semaphorin 7a exerts pleiotropic effects to promote breast tumor progression. Oncogene.

[20] Liu TJ, Guo JL, Wang HK, Xu X (2018). Semaphorin-7A contributes to growth, migration and invasion of oral tongue squamous cell carcinoma through TGF-beta-mediated EMT signaling pathway. Eur. Rev. Med. Pharm. Sci..

[21] Hu S, Liu Y, You T, Zhu L (2018). Semaphorin 7A promotes VEGFA/VEGFR2-mediated angiogenesis and intraplaque neovascularization in ApoE^−/−^ mice. Front. Physiol..

[22] Gan Y (2011). Role of semaphorin 7a signaling in transforming growth factor beta1-induced lung fibrosis and scleroderma-related interstitial lung disease. Arthritis Rheum..

[23] Costa C (2015). Expression of semaphorin 3A, semaphorin 7A and their receptors in multiple sclerosis lesions. Mult. Scler..

[24] Xie J, Wang H (2017). Semaphorin 7A as a potential immune regulator and promising therapeutic target in rheumatoid arthritis. Arthritis Res. Ther..

[25] Holmes S (2002). Sema7A is a potent monocyte stimulator. Scand. J. Immunol..

[26] Piera-Velazquez S, Jimenez SA (2019). Endothelial to mesenchymal transition: role in physiology and in the pathogenesis of human diseases. Physiol. Rev..

[27] Leask A, Abraham DJ (2004). TGF-beta signaling and the fibrotic response. FASEB J..

[28] Xie Y (2019). LMO7 is a negative feedback regulator of transforming growth factor β signaling and fibrosis. Circulation.

[29] Zhou H (2018). Activating transcription factor 3 in cardiovascular diseases: a potential therapeutic target. Basic Res. Cardiol..

[30] Tamagnone L (1999). Plexins are a large family of receptors for transmembrane, secreted, and GPI-anchored semaphorins in vertebrates. Cell.

[31] Hakanpaa L (2015). Endothelial destabilization by angiopoietin-2 via integrin β1 activation. Nat. Commun..

[32] Song X (2019). lncITPF promotes pulmonary fibrosis by targeting hnRNP-L depending on its host gene ITGBL1. Mol. Ther..

[33] Hu S (2018). Vascular semaphorin 7A upregulation by disturbed flow promotes atherosclerosis through endothelial β1 integrin. Arteriosclerosis, Thrombosis, Vasc. Biol..

[34] Pervan CL (2017). Smad-independent TGF-β2 signaling pathways in human trabecular meshwork cells. Exp. Eye Res..

[35] Dufton NP (2017). Dynamic regulation of canonical TGF-β signalling by endothelial transcription factor ERG protects from liver fibrogenesis. Nat. Commun..

[36] Song B (2019). Targeting FOXA1-mediated repression of TGF-β signaling suppresses castration-resistant prostate cancer progression. J. Clin. Invest..

[37] Morikawa M, Derynck R (2016). TGF-β and the TGF-β family: context-dependent roles in cell and tissue. Physiol. CSH Perspect. Biol.

[38] Xiang Y, Zhang Y, Tang Y, Li Q (2017). MALAT1 modulates TGF-β1-induced endothelial-to-mesenchymal transition through downregulation of miR-145. Cell Physiol. Biochem..

[39] Maleszewska M (2013). IL-1β and TGF-β2 synergistically induce endothelial to mesenchymal transition in an NFκB-dependent manner. Immunobiology.

[40] Gutiérrez-Franco A (2017). Semaphorin 7A as a potential therapeutic target for multiple sclerosis. Mol. Neurobiol..

[41] Yin X (2010). ATF3, an adaptive-response gene, enhances TGF{beta} signaling and cancer-initiating cell features in breast cancer cells. J. Cell Sci..

[42] O’Reilly MA (1992). Identification of an activating transcription factor (ATF) binding site in the human transforming growth factor-beta 2 promoter. J. Biol. Chem..

[43] Zhao W, Sun M, Li S, Chen Z, Geng D (2018). Transcription factor ATF3 mediates the radioresistance of breast cancer. J. Cell Mol. Med..

[44] Gokulnath M, Swetha R, Thejaswini G, Shilpa P, Selvamurugan N (2017). Transforming growth factor-β1 regulation of ATF-3, c-Jun and JunB proteins for activation of matrix metalloproteinase-13 gene in human breast cancer cells. Int. J. Biol. Macromol..

[45] Teasdale JE (2017). Cigarette smoke extract profoundly suppresses TNFα-mediated proinflammatory gene expression through upregulation of ATF3 in human coronary artery endothelial cells. Sci. Rep..

[46] Kawauchi J (2002). Transcriptional repressor activating transcription factor 3 protects human umbilical vein endothelial cells from tumor necrosis factor-α-induced apoptosis through down-regulation of p53 transcription. J. Biol. Chem..

[47] Nawa T (2002). Expression of transcriptional repressor ATF3/LRF1 in human atherosclerosis: colocalization and possible involvement in cell death of vascular endothelial cells. Atherosclerosis.

[48] Zhang WY (2017). HDL inhibits saturated fatty acid mediated augmentation of innate immune responses in endothelial cells by a novel pathway. Atherosclerosis.

[49] Aung HH (2016). Lipotoxic brain microvascular injury is mediated by activating transcription factor 3-dependent inflammatory and oxidative stress pathways. J. Lipid Res..

[50] De Minicis S (2013). Semaphorin 7A contributes to TGF-β–mediated liver fibrogenesis. Am. J. Pathol..

[51] Wynn TA, Ramalingam TR (2012). Mechanisms of fibrosis: therapeutic translation for fibrotic disease. Nat. Med..

[52] Almudéver P (2013). Role of tetrahydrobiopterin in pulmonary vascular remodelling associated with pulmonary fibrosis. Thorax.

