# Supplementary material for: Semaphorin 7A promotes endothelial to mesenchymal transition through ATF3 mediated TGF-β2/Smad signaling
Source: Cell Death Dis. 2020 Aug 10;11(8):695. doi: 10.1038/s41419-020-02818-x (PMC7442651; doi:10.1038/s41419-020-02818-x)
Supplement: Supplementary file 1 — supplemental table 1 [file 41419_2020_2818_MOESM1_ESM.docx]

Supplementary Table 1. Primers used for qPCR (human)

| Gene | Forward(5’-3’) | Reverse(5’-3’) |
| --- | --- | --- |
| Sema7A | GGTGCTTTTCCACGAGCCA | GTAGTTCTCGCAGTCCGTGC |
| CD31 | AACAGTGTTGACATGAAGAGCC | TGTAAAACAGCACGTCATCCTT |
| VE-cadherin | TTGGAACCAGATGCACATTGAT | TCTTGCGACTCACGCTTGAC |
| α-SMA | AAAAGACAGCTACGTGGGTGA | GCCATGTTCTATCGGGTACTTC |
| FSP-1 | GATGAGCAACTTGGACAGCAA | CTGGGCTGCTTATCTGGGAAG |
| GAPDH | CATGAGAAGTATGACAACAGCCT | AGTCCTTCCACGATACCAAAGT |
| TGF-β2 | CAGCACACTCGATATGGACCA | CCTCGGGCTCAGGATAGTCT |
| TGF-β1 | GGCCAGATCCTGTCCAAGC | GTGGGTTTCCACCATTAGCAC |
| ATF3 | CCTCTGCGCTGGAATCAGTC | TTCTTTCTCGTCGCCTCTTTTT |
| Collagen1α1 | GAGGGCCAAGACGAAGACATC | CAGATCACGTCATCGCACAAC |
| Collagen1α2 | GTTGCTGCTTGCAGTAACCTT | AGGGCCAAGTCCAACTCCTT |
| Collagen2α1 | TGGACGATCAGGCGAAACC | GCTGCGGATGCTCTCAATCT |
| Collagen3α1 | TTGAAGGAGGATGTTCCCATCT | ACAGACACATATTTGGCATGGTT |
| Fibronectin3A1 | TCTGTGCCTCCTATCTATGTGC | GAGGGACCACGACAACTCTTC |
