# Supplementary material for: Semaphorin 7A promotes endothelial to mesenchymal transition through ATF3 mediated TGF-β2/Smad signaling
Source: Cell Death Dis. 2020 Aug 10;11(8):695. doi: 10.1038/s41419-020-02818-x (PMC7442651; doi:10.1038/s41419-020-02818-x)
Supplement: Supplementary file 2 — supplemental figure legends [file 41419_2020_2818_MOESM2_ESM.docx]

**Figure S1: Generation of Sema7A-overexpressing HUVECs (Sema7A-HUVECs).** Human Sema7A overexpression plasmid construct with GFP tag (Lenti-con335-hSema7A-GFP) was transduced into HUVECs. **A**. Illustration of con335-hSema7A-GFP construct. **B.** Sema7A sequence. The sequences in red are the insert. The sequences in black are the vector. The underlines are the enzyme cleavage sites.

**Figure S2: Generation of ATF-overexpressing HUVECs (ATF3-HUVECs).** Human ATF3 overexpression plasmid construct with GFP tag (Lenti-con335-hATF3-GFP) was transduced into HUVECs and 293T. **A.** Illustration of con335-hATF3-GFP construct. **B.** ATF3 sequence. The sequences in red are the insert. The sequences in black are the vector. The underlines are the enzyme cleavage sites. **C.** ATF3 mRNA levels in ATF3-HUVECs and negative control was performed by qPCR normalized to GAPDH. Fold changes are shown. Data are mean ± SEM, N = 3, **** *P* < 0.0001.

**Figure S3: Generation of ATF3 siRNA in Lenti-con207-hATF3 transduced HUVECs**. Human ATF3 siRNA construct was transduced into HUVECs. **A.** Illustration of con207-hATF3 construct. **B.** ATF3 siRNA sequence. **C**. Cells were treated with ATF3-siRNA (ATF3-si) or empty vector, and ATF3 mRNA level was analyzed by qPCR normalized to GAPDH. Fold changes are shown. Data are mean ± SEM, N = 3, ***P* < 0.01. **D**. ATF3 protein expression were analyzed by Western blotting, normalized to tubulin and displayed as fold changes. Data are mean ± SEM, N = 3, ***P* < 0.01.

**Figure S4 A partial carotid ligation (PCL) model in mice**. **A.** Branches of the left common carotid artery (LCA), including external carotid artery (ECA), internal carotid artery (ICA) and occipital artery (OA), but not the superior thyroid artery (STA), were ligated (red lines). **B.** Ultrasound images showing flow velocity profiles. The ligation induces flow reversal (arrows) in LCA (right) during diastole. Flow in control RCA (right common carotid artery) was unchanged (left). Images are representative of the data from three mice.
