# Supplementary figures and images for: Semaphorin 7A promotes endothelial to mesenchymal transition through ATF3 mediated TGF-β2/Smad signaling
Source: Cell Death Dis. 2020 Aug 10;11(8):695. doi: 10.1038/s41419-020-02818-x (PMC7442651; doi:10.1038/s41419-020-02818-x)

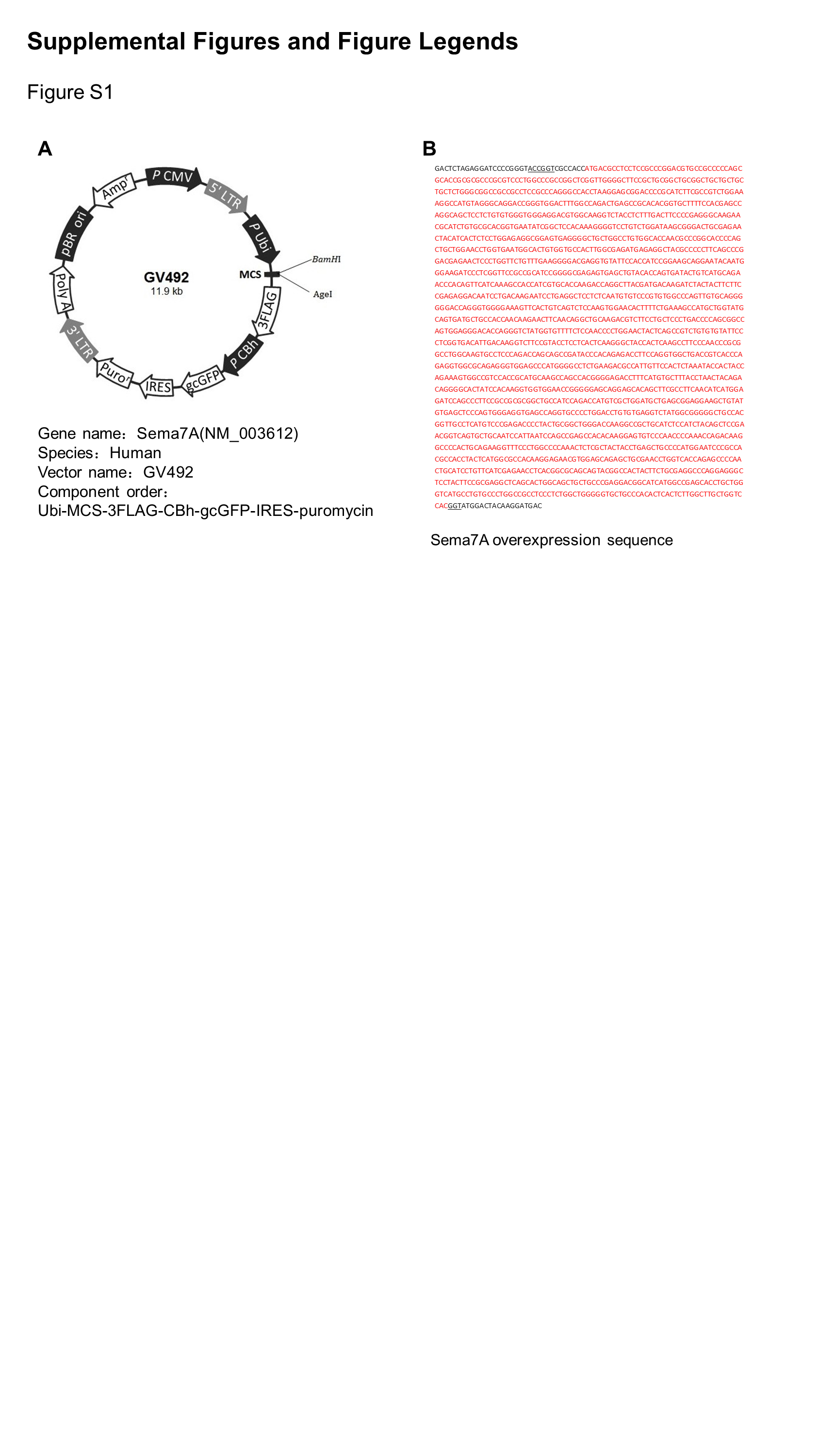

Supplement: Supplementary file 3 — supplemental figure 1 [file 41419_2020_2818_MOESM3_ESM.tif]

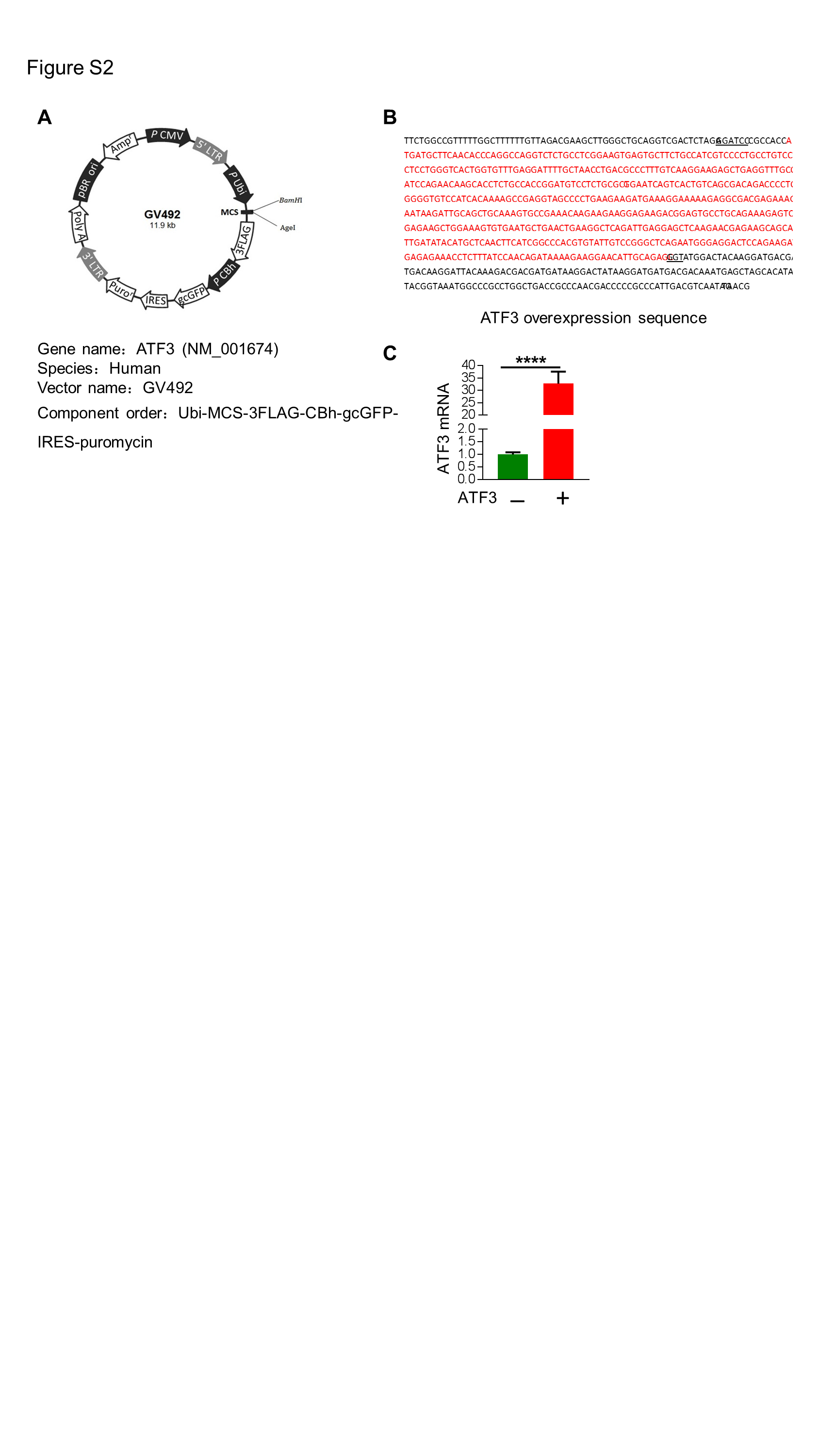

Supplement: Supplementary file 4 — supplemental figure 2 [file 41419_2020_2818_MOESM4_ESM.tif]

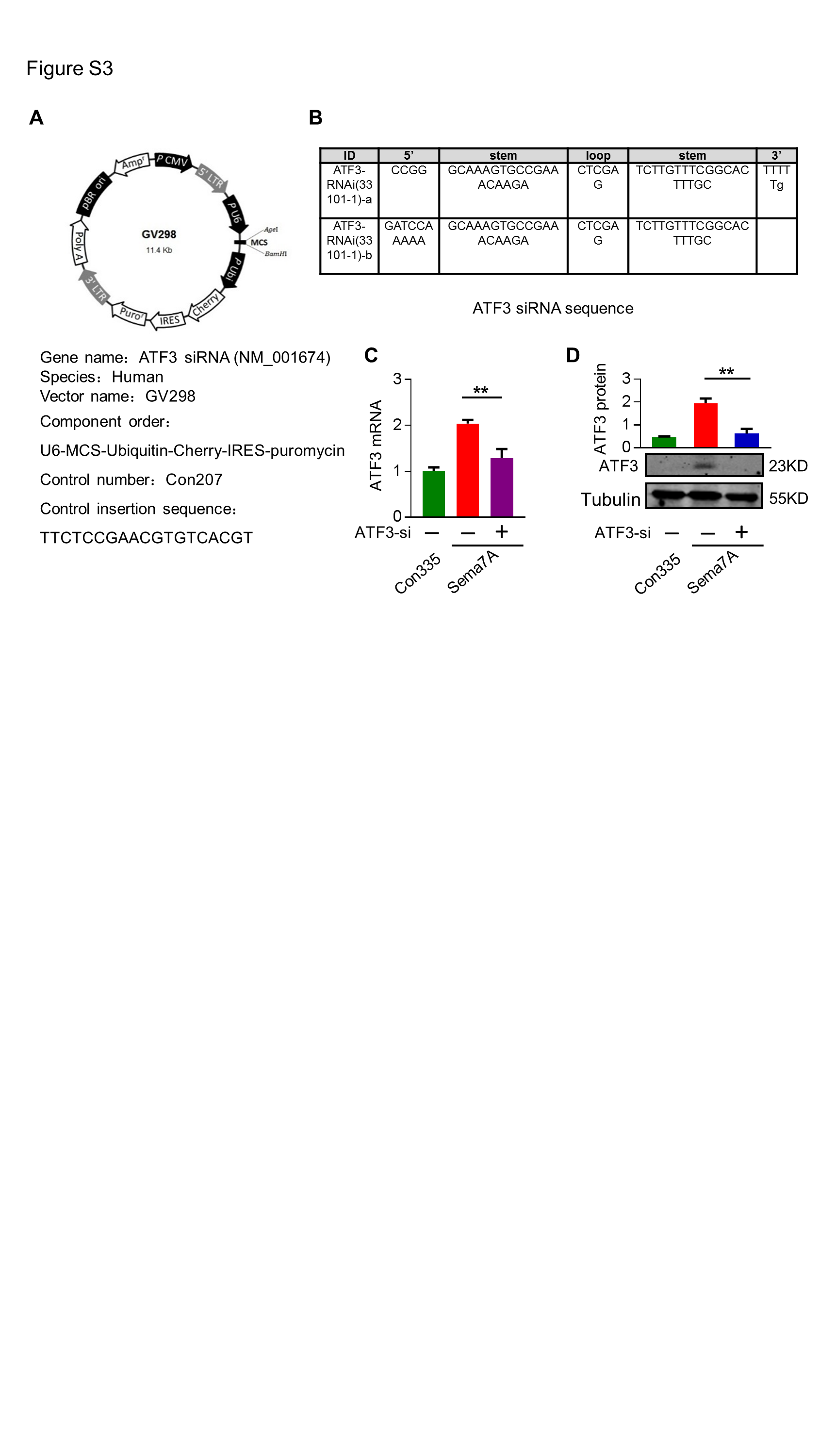

Supplement: Supplementary file 5 — supplemental figure 3 [file 41419_2020_2818_MOESM5_ESM.tif]

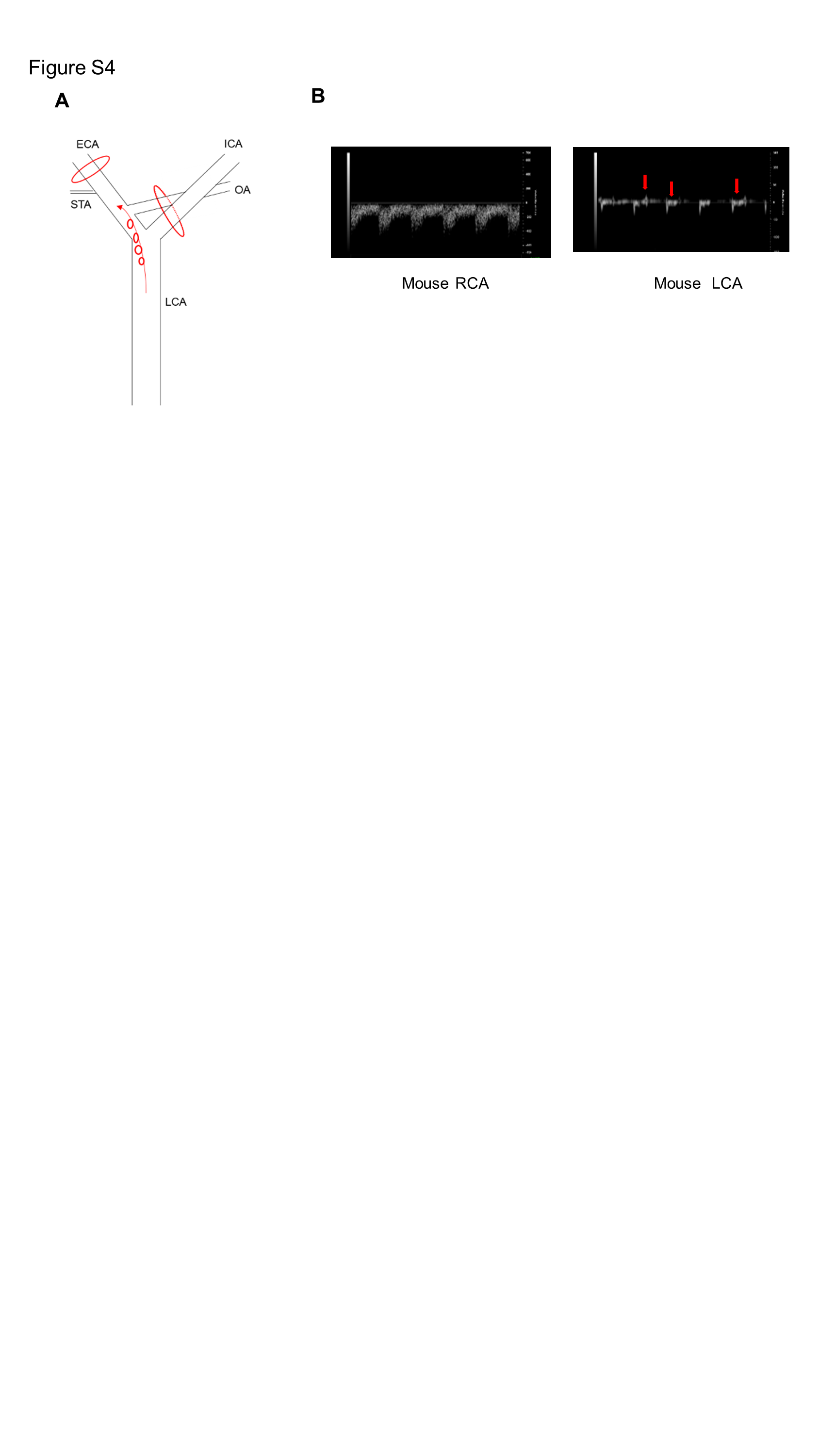

Supplement: Supplementary file 6 — supplemental figure 4 [file 41419_2020_2818_MOESM6_ESM.tif]
